# Sodium–Glucose Cotransporter-2 Inhibitor, Empagliflozin, Suppresses the Inflammatory Immune Response to Influenza Infection

**DOI:** 10.4049/immunohorizons.2300077

**Published:** 2023-12-19

**Authors:** Nicholas J. Constantinesco, Baskaran Chinnappan, Louis J. DeVito, Crystal Moras, Sashwath Srikanth, Maria de la Luz Garcia-Hernandez, Javier Rangel-Moreno, Radha Gopal

**Affiliations:** * Department of Pediatrics, University of Pittsburgh, University of Pittsburgh Medical Center, Children’s Hospital of Pittsburgh, Pittsburgh, PA; † Division of Allergy, Immunology and Rheumatology, Department of Medicine, University of Rochester, Rochester, NY

## Abstract

Influenza is a highly contagious, acute respiratory disease that causes significant public health and economic threats. Influenza infection induces various inflammatory mediators, IFNs, and recruitment of inflammatory cells in the host. This inflammatory “cytokine storm” is thought to play a role in influenza-induced lung pathogenesis. Empagliflozin is a drug primarily used to lower blood glucose in type II diabetes patients by inhibiting the sodium–glucose cotransporter-2 (SGLT-2) found in the proximal tubules in the kidneys. In this study, we have investigated the effects of empagliflozin on the pulmonary immune response to influenza infection. C57BL/6 mice (wild type) were infected with influenza A/PR/8/34 and treated with empagliflozin, and the disease outcomes were analyzed. Empagliflozin treatment decreased the expression of the inflammatory cytokines IL-1β, IL-6, and CCL2; the percentage of inflammatory monocytes and inducible NO synthase–positive macrophages; and IFN response genes Stat1 and CXCL9 during influenza infection. Further, empagliflozin treatment decreases the expression of IL-6, CCL2, and CCL5 in RAW264.7 macrophages and bone marrow–derived macrophages. However, empagliflozin treatment increased influenza viral titer during infection. Despite fostering an increased viral burden, treatment with empagliflozin decreases the mortality in wild type and high fat diet–induced atherosclerotic LDLR^−/−^ mice. Based on our findings, empagliflozin may have therapeutic implications for use in patients to prevent lung damage and acute respiratory illness.

## Introduction

Influenza A is a common viral infection that causes respiratory disease and poses a hazard to humans globally. Influenza virus causes 3–5 million infections each year, with 250,000 to 500,000 fatalities. The severity of the infection is highly variable, but nevertheless, severe cases requiring hospitalization occur annually.

The virus enters the host’s upper respiratory tract by inhaling viral particles. In response to influenza viral entry, epithelial cells induce proinflammatory cytokines and chemokines such as IL-1β, IL-6, TNFα, CCL2, CCL3, and CCL5, which recruit macrophages and neutrophils to the site of infection to help control the virus. These immune cells induce NO and reactive oxygen species, which increases lung injury. TLRs and the retinoic acid–inducible gene (RIG-I) recognize viral pathogen-associated molecular patterns upon viral entry, activating downstream NF-κB, MAPK, and IFN regulatory factor 3 signaling pathways and inducing inflammatory cytokines and IFNs to control the virus (1, 2). The inflammatory response may become uncontrollable, culminating in a cytokine storm that can cause severe illness. There are limited therapeutics for influenza other than supportive care. Because most of the damage is host-driven, targeting inflammation is a strong candidate for reducing disease severity and hospitalization.

Type I and III IFNs play a key role in reducing pathogen infection by activating innate immune responses (3, 4). Type II IFNs, such as IFN-γ produced by T and NK cells, promote anti-viral immunity by regulating innate and adaptive immune responses (5). Type I, II, and III IFNs inhibit viral replication by inducing IFN-stimulated genes (ISGs), such as MX dynamin-like GT Pase1 (Mx1) and 2′,5′-oligoadenylate synthase–like protein (OASL2), via JNK-dependent phosphorylation of Stat1 and Stat2 to promote the antiviral response (6).

SGLT-2 inhibitors are a prescribed medication commonly used by patients with type II diabetes. Empagliflozin, an SGLT-2 inhibitor, can help lower blood glucose levels by decreasing glucose reabsorption in the proximal convoluted tubules of the nephron and increasing glucose excretion in the urine. Empagliflozin has a diuretic property that helps remove excess urine from the body and a natriuretic property that helps reduce sodium in the blood, thus lowering blood pressure. Previously, it has been shown that empagliflozin improved respiratory function in reperfusion-induced lung injury decreased myocardial inflammation and is used to prevent cardiovascular death (7–9). Similarly, other studies have shown the beneficial effects of empagliflozin treatment in pulmonary hypertension and chronic obstructive pulmonary disease (5, 10). Studies show that its anti-inflammatory effects help in reducing oxidative stress and stimulating anti-inflammatory macrophages and anti-inflammatory cytokines (11–13).

Because the host immune system-induced cytokine storm causes severe pathology during severe influenza infection, we hypothesized that empagliflozin may reduce the influenza-induced proinflammatory response, thereby decreasing the severity of disease and promoting health and recovery. In this paper, we examined the effect of empagliflozin on the inflammatory mechanisms involved during influenza infection in both in vivo and in vitro models and show the possible mechanisms involved in those responses.

## Materials and Methods

### Animal experiments

C57BL/6 mice (6–8 wk old) were purchased from Taconic Farms, and LDLR^−/−^ mice (6–8 wk old) were bought from The Jackson Laboratory. The mice were kept under pathogen-free conditions at the University of Pittsburgh Medical Center Children’s Hospital of Pittsburgh. The mice used for these studies were age- (6–8 wk) and sex-matched. LDLR^−/−^ mice (8–10 wk old) were fed a high-fat diet (HFD) having 42% kcal fat (Teklad) for 11 wk. Mouse experiments were approved by the University of Pittsburgh Institutional Animal Care and Use Committee.

### Influenza viral infection

Influenza A PR/8/34 H1N1 virus was propagated and titrated in Madin Darby canine kidney cells (14, 15). The mice were treated with influenza A/PR/8/34 H1N1 (1000 PFU) or PBS by oropharyngeal aspiration (15). The mice were treated orally with empagliflozin (10 mg/kg body weight) or a vehicle (DMSO) daily for 7 or 14 d, and the mice were sacrificed. The empagliflozin was dissolved in DMSO (50 mg/kg body weight). The drug was diluted in PBS and administered orally. For vehicle control, the DMSO was dissolved in PBS and administered. The viral burden was accessed by gene expression analysis of influenza viral matrix protein using forward primer: 5′-GGACTGCAGCGTAGACGCTT-3′; reverse primer: 5′-CATCCTGTTGTATATGAGGCCCAT-3′; Probe: 5′-/56-FAM/CTCAGTTAT/ZEN/TCTG CTG GTGCACTTGCCA/3IABkF Q/−3′; and plaque assay (16, 17).

### Analysis of lung inflammation

Mouse lungs were processed as described before (18–21). Lungs were lavaged with 1 ml PBS to collect the bronchoalveolar lavage (BAL). The middle and caudal lung lobes were used for RNA analysis using an RNA isolation kit (Agilent Technologies, Santa Clara, CA). RNA was converted to cDNA using reverse transcription supermix (Bio-Rad Laboratories, Hercules, CA). Gene expression was analyzed by RT-PCR using TaqMan probes and primers (Thermo Fisher Scientific) and predesigned primer probes (Integrated DNA Technologies). The upper right lung lobe homogenate was used for cytokine analysis by Bio-plex multiplex immunoassay (Bio-Rad). The left lung lobe was collected in 10% formalin, 5-μm tissue sections were cut from paraffin-embedded blocks, and the lung inflammatory parenchymal and peribronchial scores were analyzed as described before (22, 23).

### Detection of inducible NO synthase and CD206-expressing macrophages

Lung tissue sections were stained with a goat anti-mouse CD206 (catalog no. AF2535, RRID:AB_2063012, R&D Systems, Minneapolis, MN), rabbit anti-mouse inducible NO synthase (iNOS) (catalog no. NB300-605, RRID:AB_10002794, Novus Biologicals, Littleton, CO), and rat-anti mouse F4/80 (clone Cl:A3-1, RRID:AB_323279, Bio-Rad) to detect M1 and M2 macrophages. Primary Abs were visualized by incubation with the following secondary Abs: (A11057, RRID: AB_142581, Thermo Fisher Scientific, Waltham, MA), Alexa Fluor 488 donkey anti-rabbit IgG (711-546-152, RRID:AB_2340619, Jackson ImmunoResearch Laboratories, Bar Harbor, ME), and Alexa Fluor 647 donkey anti-rat IgG (712-606-153, RRID:AB_2340696, Jackson ImmunoResearch Laboratories).

Briefly, 5-μm paraffin lung sections were incubated at 60°C for deparaffinization. Tissue sections were transferred to xylenes and gradually hydrated by sequential transfer into alcohol, 95% alcohol, 70% alcohol, and water. Sections were then immersed in Ag retrieval solution (S1699, Agilent DAKO, Santa Clara, CA) and boiled for 30 min. Nonspecific binding was blocked by incubating tissue sections with 5% normal donkey serum (Jackson ImmunoResearch Laboratories) at room temperature for 30 min in a humid chamber. Immediately after removing the blocking solution, primary Abs were added to the tissue sections. Slides were incubated overnight with the primary Abs at room temperature. Tissue sections were washed with PBS and secondary Abs were incubated for 1 h at room temperature. Finally, the tissues were washed with PBS and mounted with Vectashield antifade mounting medium with DAPI (H-1200, Vector Laboratories, Newark, CA). Mosaic images were taken with a Zeiss Axioplan 2 microscope and collected with a Hamamatsu camera. F4/80^+^ macrophages expressing CD206 or iNOS were counted with a tool of the Zeiss Axioplan microscope in three random 200× fields found in the lung parenchyma of individual lung sections.

### Flow cytometry

Mouse lungs were collected, dissected into small pieces, digested at 37°C in 1 mg/ml of collagenase medium for 1 h, passed through 70-μm filters, and stained for flow cytometry analysis to determine cellular subsets as described before (20). The single cells were stained with Abs specific to CD45 (30-F11), CD11b (M1/70), CD11c (N418), F4/80 (BM/8), Ly6C (AL-21), CD64 (X54-5/7.1), and Siglec F (1RNM44N). Data were collected with a Cytek Aurora flow cytometer, and the frequency of the cell types was analyzed with FlowJo software.

### Cell culture studies

RAW 264.7 macrophages and C10 lung epithelial cells were kept and cultured in DMEM supplemented with FBS (10%), penicillin (100 IU/ml), and streptomycin (10 ug/ml) in a CO_2_ incubator (5% CO_2_, 37°C). Femur and tibia were isolated from C57BL/6 mice and cultured with GM-CSF for 1 wk to generate bone marrow–derived macrophages (BMDMs). The adherent cells were isolated, counted, and plated for 24 h. Empagliflozin was dissolved in DMSO, and a stock solution was made. The cells were treated with either empagliflozin (80 μM) or a vehicle (DMSO) or with influenza (1 multiplicity of infection [MOI]).

The cell supernatants from cell experiments were collected for protein, cytokine, and chemokine analysis. The cell lysate was collected in RNA lysis buffer for gene expression analysis. RNA analysis using the Aurum total RNA isolation kit (Bio-Rad). RNA was converted to cDNA using reverse transcription supermix (Bio-Rad). Gene expression was analyzed by quantitative RT-PCR using TaqMan probes and primers (Thermo Fisher Scientific) and predesigned primer probes (Integrated DNA Technologies). The cell supernatants were analyzed by ELISA by using the duo set ELISA kits (R&D Systems, Minneapolis, MN).

### Statistical analysis

The data were analyzed as means ± SEM. The data were analyzed using GraphPad Prism software. Significance was tested by unpaired *t* test or one-way ANOVA followed by Tukey’s post hoc test. All studies were repeated two to three times, and the data were combined unless otherwise described.

### Data availability

The datasets generated during and/or analyzed during the current study are available from the corresponding author on reasonable request.

## Results

### Empagliflozin treatment decreases the expression of proinflammatory cytokines and recruitment of inflammatory monocytes during influenza infection

**FIGURE 1.**
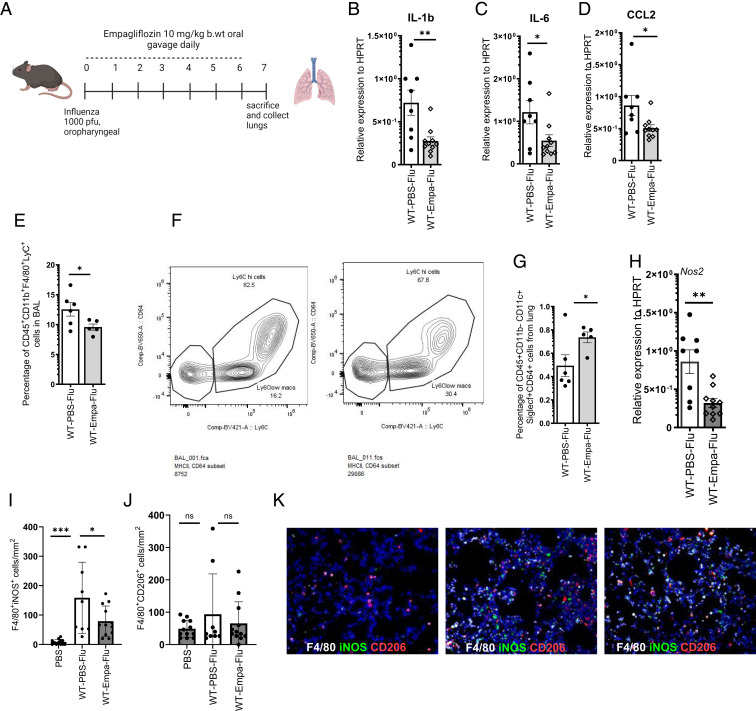
Empagliflozin suppresses the expression of proinflammatory cytokines and chemokines in the lungs. (**A**) C57BL/6 mice were treated with PBS or 10^3^ PFU of influenza A PR/8/34 on day 0 and were treated with either DMSO vehicle or empagliflozin for 7 d. (**B**–**D**) Relative expressions of IL-1b, IL-6, and CCL2 in the lungs were measured by RT-PCR (*n* = 8–10). (**E** and **F**) The percentage of CD45^+^CD11b^+^F4/80^+^LyC^+^ cells. (**G**) CD45^+^CD11b^−^CD11c^+^SiglecF^+^CD64^+^ cells from BAL by flow cytometry (*n* = 5–6). (**H**) Relative expression of NOS2 was measured by RT-PCR (*n* = 8–10). (**I**–**K**) F4/80^+^ macrophages expressing CD206 or iNOS were counted in three 200× images (I and J) and the representative images (K). The data are represented as means ± SEM. Significance was tested by unpaired *t* test or one-way ANOVA. Each experiment was independently performed two or more times, and representative data are shown from combined (B–D and H) or independent (E–G and I–K). *p < 0.05, **p < 0.01, ***p < 0.001, ****p < 0.0001. b.wt, body weight; ns, not significant; WT, wild type.

To determine the effect of empagliflozin on the immune response during influenza infection, we infected C57BL/6 mice with influenza, treated them with empagliflozin, and determined the expression and the levels of inflammatory cytokines, chemokines, and frequencies of immune cells on day 7 after influenza infection (Fig. 1A). We found that empagliflozin decreases the expression of IL-1β, IL-6, and CCL2 when compared with vehicle-treated controls (Fig. 1B–D). Next, we analyzed the protein levels of IL-1β, IL-6, and CCL2 and found no significant differences between empagliflozin treatment and vehicle controls (Supplemental Fig. 1). Further, we found no significant differences in parenchymal and peribronchial lung inflammatory histological scoring (Supplemental Fig. 2). However, we found a decrease in the frequency of inflammatory monocytes from BAL (Fig. 1E, 1F). These data suggest that empagliflozin treatment suppresses the proinflammatory immune response without affecting the pathology during influenza infection.

Earlier studies have shown that ∼90% of alveolar macrophages are depleted during the first week of influenza infection (24). In this study, we found that the percentage of alveolar macrophages significantly increased in response to empagliflozin treatment (Fig. 1G). These data suggest that empagliflozin treatment promotes maintenance of homeostatic alveolar macrophages in the lung.

It has been shown that empagliflozin reduces reactive oxygen species and improves mitochondrial function (25). In our study, we found that empagliflozin reduces NOS2 expression, suggesting that empagliflozin reduces the induction of reactive nitrogen species, thereby reducing the mitochondrial oxidative stress (Fig. 1H). Further, we found that the iNOS^+^F4/80^+^ macrophages were decreased in mice treated with empagliflozin when compared with vehicle controls (Fig. 1I).

M2 macrophages are known to be anti-inflammatory and proresolving; thus we analyzed whether the empagliflozin-mediated pulmonary immune response skews toward an M2 macrophage phenotype (20). CD206 is shown to be a marker for M2 macrophages (26–28). Therefore, we analyzed whether empagliflozin influences the frequency of M2 macrophages. We found that the frequency of CD206^+^ macrophages was not affected by empagliflozin treatment during influenza infection (Fig. 1J). Also, we observed no differences in the expression of anti-inflammatory cytokines IL-4, IL-10, and TGFβ between empagliflozin-treated and vehicle control groups (Supplemental Fig. 3). These data suggest that the empagliflozin treatment suppresses the M1 macrophages without affecting the frequency of M2 macrophages or anti-inflammatory cytokines during influenza infection.

**FIGURE 2.**
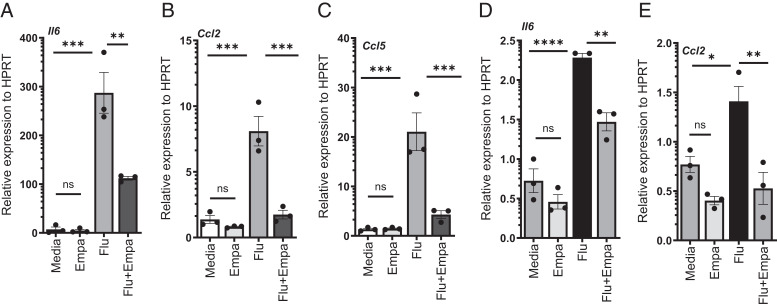
Empagliflozin suppresses the expression of proinflammatory cytokines and chemokines in BMDMs and RAW 264.7 macrophages. BMDMs or RAW264.7 Macrophages were treated with PBS or 1 MOI of influenza A PR/8/34 and either DMSO vehicle or empagliflozin for 24 h. Relative expressions of IL-6, CCL2, and CCL5 from BMDMs (**A**–**C**) and IL-6 and CCL2 (**D** and **E**) were measured by RT-PCR. The data are represented as means ± SEM. The cells were treated with triplicates or quadruplicates. Significance was tested by unpaired *t* test or one-way ANOVA. Each experiment was independently performed two or more times, and representative data are shown. *p < 0.05, **p < 0.01, ***p < 0.001, ****p < 0.0001. Empa, empagliflozin; ns, not significant.

Next, we examined whether empagliflozin suppresses the proinflammatory immune response in macrophages directly. RAW264.7 or BMDMs were treated with empagliflozin and/or influenza, and proinflammatory cytokine expression was analyzed. We found that empagliflozin treatment decreased the expression of IL-6, CCL2, and CCL5 in BMDMs (Fig. 2A–C) and IL-6 and CCL2 in RAW264.7 macrophages (Fig. 2D, 2E) during influenza infection. These data suggest that the empagliflozin treatment directly suppresses the inflammatory immune responses in macrophages during influenza infection.

### Empagliflozin treatment decreases TLR3 expression during influenza infection

**FIGURE 3.**
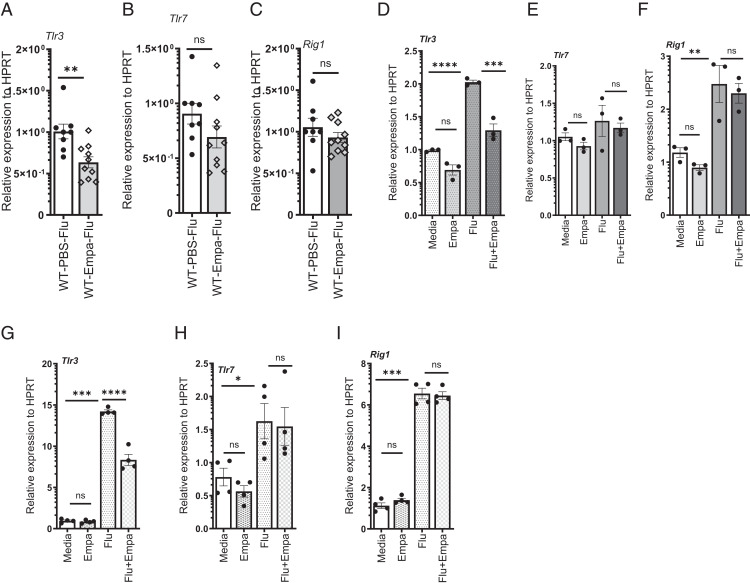
TLR3 expression is decreased in mice, RAW264.7 macrophages, and lung epithelial C-10 cells treated with empagliflozin. C57BL/6 mice were treated with PBS or 10^3^ PFU of influenza A PR/8/34 on day 0 and were treated with either DMSO vehicle or empagliflozin for 7 days. (**A**–**C**) Relative expressions of TLR3. TLR7, and RIG-I in the lungs were measured by RT-PCR (*n* = 8–10). (**D**–**F**) RAW264.7 Macrophages were treated with PBS or 1 MOI of influenza A PR/8/34 and either DMSO vehicle or empagliflozin for 24 h, and the expression of TLR3, TLR7, and RIG-I was measured by RT-PCR. (**G**–**I**) C10 cells were treated with PBS or 1 MOI of influenza A PR/8/34 and either DMSO vehicle or empagliflozin for 24 h, and the expression of TLR3 (G), TLR7 (H), and RIG-I (I) was analyzed. The data are represented as means ± SEM. The cells were treated with triplicates or quadruplicates. Significance was tested by unpaired *t* test or one-way ANOVA. Each experiment was independently performed two or more times, and representative data are shown from combined (A–C) or independent (D–I). *p < 0.05, **p < 0.01, ***p < 0.001, ****p < 0.0001. Empa, empagliflozin; ns, not significant.

Next, we proposed that the decrease in the proinflammatory cytokine response may be due to a decrease in the innate viral-sensing mechanisms. TLR3, TLR7, TLR8, and RIG-I are known to be involved in sensing influenza viral Ags and activating downstream signaling pathways (1, 29). To address whether the decrease in the proinflammatory cytokine responses may be due to a defect in innate immune sensing, we analyzed the expression levels of TLR3, TLR7, and RIG-I from lung samples from mice treated with empagliflozin, as well as vehicle-treated controls. We found that empagliflozin decreased the expression of TLR3 in the lung (Fig. 3A). However, there were no significant differences between the treatment and control groups in the expression of TLR7 and RIG-I (Fig. 3B, 3C). Further, RAW264.7 macrophages treated with empagliflozin also showed a decrease in TLR3 expression but showed no difference in the expression of TLR7 and RIG-I between empagliflozin and vehicle control groups (Fig. 3D–F). These data suggest that the empagliflozin-induced suppression of proinflammatory response might be due to a decrease in TLR3-sensing mechanisms.

Because lung epithelial cells are the first to meet the influenza virus, we also examined the effect of empagliflozin treatment on the expression of TLR3, TLR7, and RIG-I in lung epithelial C10 cells. Similarly, to macrophages, empagliflozin treatment decreased the expression of TLR3 but had no effect on the expression of TLR7 and RIG-I between empagliflozin and vehicle control groups (Fig. 3G–I). Next, we analyzed whether the decreased TLR3 response affects the induction of proinflammatory cytokines. As expected, we found a decrease in the expression and protein levels of CCL5 in C10 cells (Supplemental Fig. 4) in response to empagliflozin treatment. These data suggest that empagliflozin decreases the TLR3-mediated innate immune-sensing mechanism, thereby decreasing the induction of chemokines.

### Empagliflozin treatment suppresses IFN response genes and increases the influenza viral burden

**FIGURE 4.**
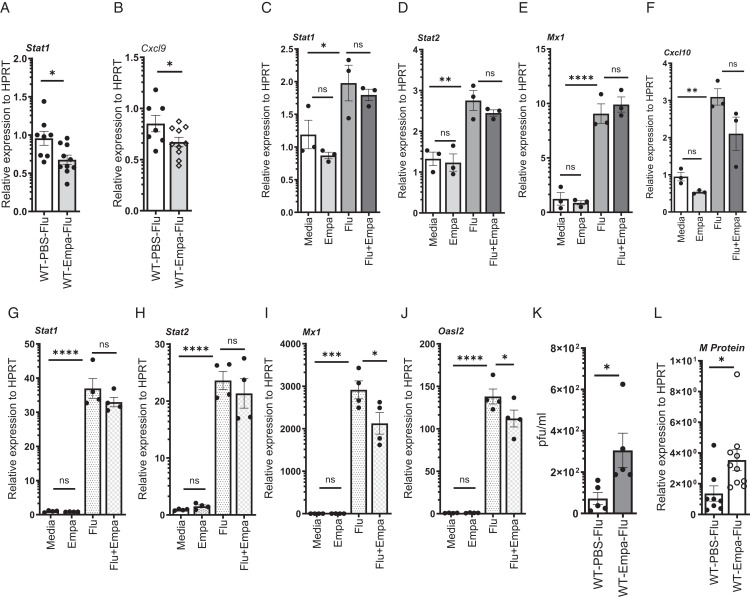
Empagliflozin decreases the IFN response genes and increases the viral titer during influenza infection. C57BL/6 mice were treated with PBS or 10^3^ PFU of influenza A PR/8/34 on day 0 and were treated with either DMSO vehicle or empagliflozin for 7 days. (**A** and **B**) Relative expressions of Stat1 and CXCL9 were analyzed by RT-PCR (*n* = 8–10). (**C**–**F**) RAW264.7 macrophages were treated with PBS or 1 MOI of influenza A PR/8/34 and either DMSO vehicle or empagliflozin for 24 h, and the expression of Stat1, Stat2, Mx1, and OASL2 was analyzed by RT-PCR. C10 cells were treated similarly and measured the expression of Stat1, Stat2, Mx1, and OASL2 (**G**–**J**). The cells were treated with triplicates or quadruplicates. C57BL/6 mice were treated with PBS or 10^3^ PFU of influenza A PR/8/34 on day 0 and were treated with either DMSO vehicle or empagliflozin for 7 days. (**K**) The plaque assay was carried out from the lung samples (*n* = 5). (**L**) Relative expressions of viral M protein in the lungs were measured by RT-PCR (*n* = 8–10). The data are represented as means ± SEM. Significance was tested by unpaired *t* test or one-way ANOVA. Each experiment was independently performed two or more times, and representative data are shown from combined (A, B, and L) or independent (C–J). *p < 0.05, **p < 0.01, ***p < 0.001, ****p < 0.0001. Empa, empagliflozin; ns, not significant; WT, wild type.

Type I, II, and III IFNs play a significant role in controlling the viral burden by induction of ISGs through activation of Stat1 and Stat2 signaling pathways (3, 4). Therefore, we analyzed the expression of IFN response genes in mice treated with empagliflozin during influenza infection. We found a decrease in the expression of Stat1 and CXCL9 in empagliflozin-treated mice when compared with vehicle controls, suggesting that empagliflozin may interfere with the Stat1 signaling pathway, thereby decreasing the induction of CXCL9 (Fig. 4A, 4B).

Next, we examined whether empagliflozin affects the direct influenza exposure-induced induction of IFN response genes in macrophages and epithelial C10 cells. We found no differences in the expression of Stat1, Stat2, Mx1, Oasl2, and CXCL10 in macrophages between empagliflozin and vehicle control groups during influenza exposure (Fig. 4C–F). Furthermore, we analyzed the same set of genes in epithelial C10 cells treated with empagliflozin and found no differences in the expression of Stat1 and Stat2 between empagliflozin and control groups (Fig. 4G, 4H). However, we found a decrease in the expression of Mx1 and Oasl2 in the empagliflozin treatment group when compared with vehicle controls during influenza infection (Fig. 4I, 4J). These data suggest that empagliflozin suppresses the induction of IFN response genes in lung epithelial cells.

Next, we found whether the decreased induction of ISGs had an impact on influenza viral control in mice. We analyzed the expression of influenza viral M protein and infectious viral particles by plaque assay. We found an increase in the expression of influenza viral M protein and more plaques in the lungs from empagliflozin treatment when compared with vehicle-treated controls (Fig. 4K, 4L). These data suggest that the decreased ISGs resulting from treatment with empagliflozin may affect viral control during influenza infection.

**FIGURE 5.**
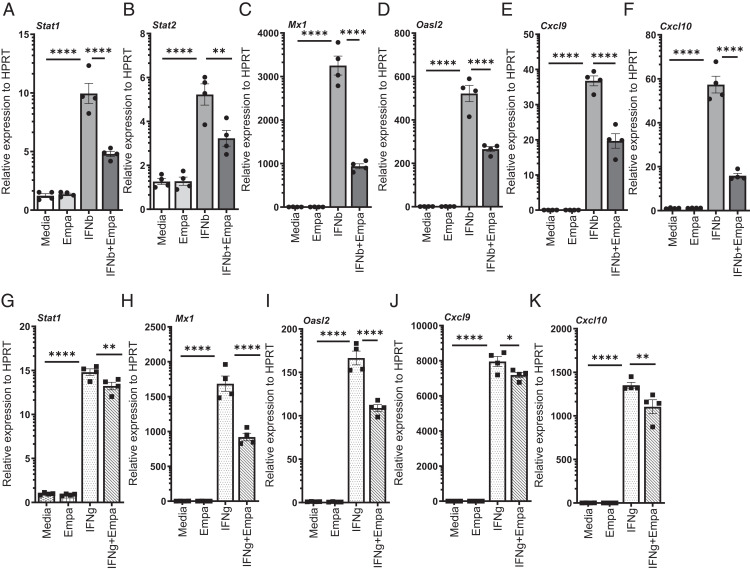
Empagliflozin decreases the IFN response genes in response to IFNβ and IFNγ treatment in RAW264.7 cells. RAW264.7 cells were treated with IFNβ (**A**–**F**) or IFNγ (**G**–**K**) and either DMSO vehicle or empagliflozin for 24 h, and the expression of Stat1, Stat2, Mx1, OASL2, CXCL9, and CXCL10 was analyzed by RT-PCR. The data are represented as means ± SEM. The cells were treated with triplicates or quadruplicates. Significance was tested by unpaired *t* test or one-way ANOVA. Each experiment was independently performed two or more times, and representative data are shown. *p < 0.05, **p < 0.01, ***p < 0.001, ****p < 0.0001. Empa, empagliflozin.

Next, we analyzed whether empagliflozin influences ISG induction by direct IFN exposure in macrophages and epithelial cells. We treated the RAW264.7 macrophages with type I (IFNβ) and type II (IFNγ) and measured the IFN response genes. The expressions of Stat1, Stat2, Mx1, Oasl2, Cxcl9, and Cxcl10 were substantially upregulated in response to IFNβ treatment. However, we found that after treatment with IFNβ in conjunction with empagliflozin, there was decreased expression of these genes (Fig. 5A–F). Similarly, the expressions of Stat1, Mx1, Oasl2, Cxcl9, and Cxcl10 were significantly decreased in response to empagliflozin treatment during IFNγ exposure (Fig. 5G–K). These results suggest that empagliflozin suppresses the ISGs induced by type I and type II IFNs in macrophages, and the influenza-induced IFN-mediated suppression of ISGs may be a mechanism that contributes to increased viral load during influenza infection.

### Empagliflozin treatment decreases the mortality rate during the influenza infection in HFD-induced atherosclerosis

**FIGURE 6.**
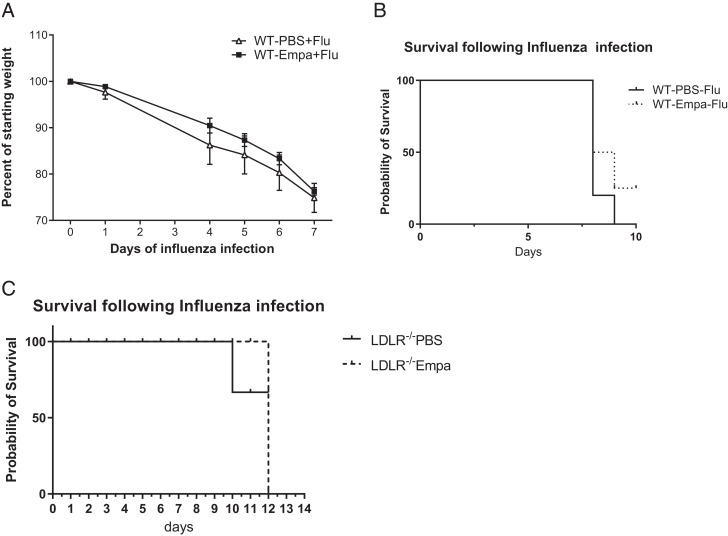
Empagliflozin treatment increases the recovery of body mass and survival following influenza infection. (**A**) C57BL/6 mice were treated with PBS or 10^3^ PFU of influenza A PR/8/34 on day 0 and were treated with either DMSO vehicle or empagliflozin for 6 days, and the percentage of weight loss was analyzed (*n* = 4–5). (**B** and **C**) C57BL/6 mice were treated with PBS or 10^4^ PFU of influenza A PR/8/34 on day 0 and were treated with either DMSO vehicle or empagliflozin (B), and the survival was analyzed daily (C). HFD-fed LDLR^−/−^ mice were treated with PBS or 7.5 × 10^2^ PFU of influenza A PR/8/34 on day 0 and were treated with either DMSO vehicle or empagliflozin, and the survival was analyzed (0–14 days) (*n* = 4). The data are represented as means ± SEM. Significance was tested by unpaired *t* test or one-way ANOVA. Each experiment was independently performed two or more times, and representative data are shown. *p < 0.05, **p < 0.01. Empa, empagliflozin.

Next, we analyzed whether the increase in influenza viral burden and decreased proinflammatory response have an impact on the outcome of influenza infection. We analyzed the clinical signs including weight loss, physical activity, and the hunching of the backs (30). The control mice become more lethargic from day 6 onwards because they do not move in their cage and do not resist being handled (data not shown). They also have more pronounced hunching of their backs on day 7 compared with empagliflozin-treated mice. However, there were no significant differences in weight loss between vehicle control and empagliflozin-treated mice during influenza infection (Fig. 6A).

Next, we analyzed whether empagliflozin affects survival during a high-viral dose influenza infection. We found that 20% of mice survived on day 8 from the empagliflozin-treated group when compared with the vehicle control group (Fig. 6B). These data suggest that the empagliflozin treatment improves clinical outcomes and survival during severe influenza infection.

Previously it has been shown that HFD increases the inflammatory immune response during influenza infection and influenza infection exacerbates atherosclerosis (31). Therefore, we analyzed whether empagliflozin treatment influences the weights in HFD-induced atherosclerosis in LDLR^−/−^ mice during influenza infection. We found that the empagliflozin treatment decreases mortality during influenza infection when compared with vehicle control-treated HFD mice (Fig. 6C). Furthermore, the mice treated with a vehicle control were more lethargic and had ruffled fur during the experiment compared with empagliflozin-treated mice (data not shown). These data suggest that empagliflozin helps promote both recovery and better clinical outcomes after influenza infection in HFD-induced atherosclerotic mice.

## Discussion

Upon viral entry, epithelial cells and other immune cells induce various cytokines and chemokines that attract macrophages and neutrophils. These immune cells induce NO and reactive nitrogen species, which increase lung injury. Inflammatory cytokines may enter the tissue through lung leak and cause inflammatory cell migration from the tissue into the circulatory system, resulting in an inflammatory cytokine storm (32, 33). In this study, we found that empagliflozin decreases proinflammatory cytokine responses. Because cytokine storms are the key mechanisms involved in pathology, suppression of the immune response may prove beneficial.7

Previously it has been shown that SGLT-2 inhibitors help to control inflammasome activation and then reduce the secretion of IL-1β in macrophages from diabetic patients with cardiovascular risk (34). It is known that influenza activates the NLRP3 inflammasome during infection and increases influenza-induced disease severity. In our study, we found that empagliflozin suppresses the induction of IL-1β during pulmonary infection. These data suggest that empagliflozin may inhibit IL-1β induction and may ultimately play a role in reducing IL-1β.

It has been previously shown that empagliflozin suppresses LPS-induced NF-κB, MAPK, and JAK/Stat signaling pathways in RAW macrophages, thereby decreasing proinflammatory cytokines and chemokines (12). In our study, the decrease in the expression of inflammatory cytokines and chemokines such as IL-1β, IL-6, and CCL2 may be due to suppression of NF-κB and MAPK signaling pathways. Decreased expression of the IFN response genes Stat1, CXCL9, and CXCL10 may be due to suppression of the JAK/Stat signaling pathways in response to empagliflozin treatment during influenza infection.

Epithelial innate sensing is important for the innate immune response. TLR3 signaling is crucial in eliciting the innate immune response through activation of signaling by Toll–IL receptor domain–containing adapter protein–inducing IFN-β and downstream NF-κB signaling pathway that leads to the production of proinflammatory cytokines and IFNs. In our study, we found the expression of TLR3 is decreased by empagliflozin. This effect may be due to empagliflozin acting on these signaling cascades. The decrease in immune sense may be related to the increase in influenza viral burden seen. Previously, it has been shown that TLR3-deficient mice have a 9-fold increase in viral burden (35). However, TLR7 and RIG-I, are not affected in response to empagliflozin treatment. Therefore, the viral immune sensing mechanism may not be completely compromised.

Type I and type III IFN signaling are crucial in controlling the influenza virus through ISG expression. The expression of Stat1 and CXCL9 decreased significantly in response to empagliflozin treatment, according to our findings. This is consistent with the research findings of Giannattasio et al. (36), who found that empagliflozin reduces CXCL10 levels and Stat1 phosphorylation in cardiomyocytes kept in a Th1 (IFNγ/TNFα) environment. Similarly, we found that the empagliflozin suppresses Stat1, Mx1, Oasl2, CXCL9, and CXCL10 following IFNβ and IFNγ exposure. These data suggest that the empagliflozin-mediated decrease in ISGs may be one of the possible mechanisms involved in the increased viral burden during influenza infection.

It has been shown that ∼90% of the alveolar macrophages are depleted during the first week of influenza infection (24). The increase in the frequency of alveolar macrophages due to empagliflozin treatment may suggest a repair mechanism. Studies have shown that M2 macrophages are upregulated in response to empagliflozin treatment in diet-induced obese mice (37). However, in our studies, empagliflozin treatment during influenza infection resulted in the suppression of M1 macrophages. However, the frequency of CD206-positive macrophages was not altered in response to empagliflozin treatment during infection. All analyses were carried out with samples collected on day 7 post–influenza infection, which is during peak infection, when clinical symptoms are observed. Empagliflozin may help increase the presence of M2 macrophages during the recovery phase (>9 d) of influenza infection.

After influenza viral entry, the virus multiplies during the early phase of infection. Shortly after, the host immune response plays a significant role in the disease’s pathogenesis. In our study, empagliflozin treatment increases the viral burden during infection. Despite reduced mRNA expression of inflammatory cytokine responses, there were no differences in weight loss, levels of inflammatory cytokines, and histological scoring between mice treated with empagliflozin or a vehicle control. It is possible this is due to a balanced effect between increased viral activity and a decrease in the inflammatory immune response due to empagliflozin treatment. However, empagliflozin decreases the mortality of influenza infection in HFD-induced atherosclerosis. Also, empagliflozin treatment reduces clinical symptoms during the influenza infection in these models (30). Based on our findings, empagliflozin may have therapeutic implications for use in patients to prevent lung damage and acute respiratory illness.

## Supplementary Material

Supplemental Figures 1 (PDF)
