# Supplementary material for: Sodium–Glucose Cotransporter-2 Inhibitor, Empagliflozin, Suppresses the Inflammatory Immune Response to Influenza Infection
Source: Immunohorizons. 2023 Dec 19;7(12):861–71. doi: 10.4049/immunohorizons.2300077 (PMC10759161; doi:10.4049/immunohorizons.2300077)
Supplement: Supplemental Figures 1 (PDF) [file IH_2300077_Supplemental_1.pdf]

Supplementary Figure-1

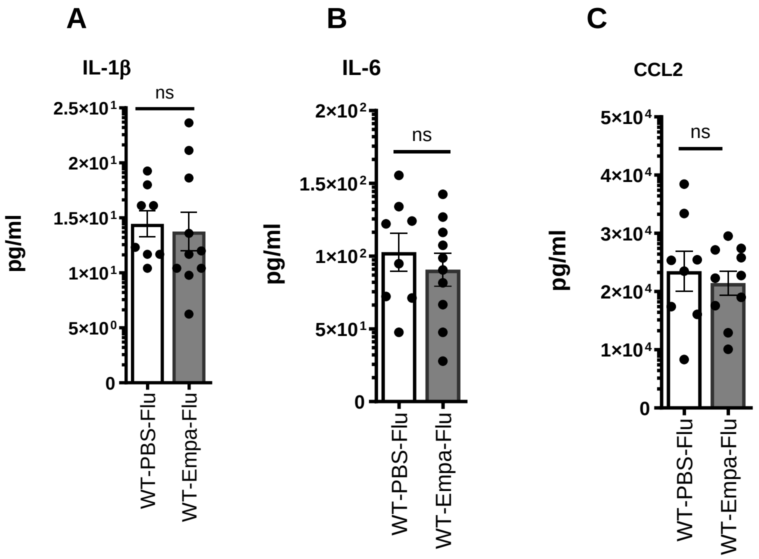

Supplementary Figure-1. Empagliflozin does not affect the levels of pro-inflammatory cytokines.

C57BL/6 mice were treated with PBS or 10^3 PFU of influenza A PR/8/34 on day 0 and were treated with either DMSO vehicle or empagliflozin for 7 days. The levels of IL-1β, IL-6, and CCL2 were measured by Bio-Plex Multiplex Immuno assay (A-C). Data are represented as mean±SEM. Significance was tested by unpaired t-test. *p < 0.05, **p < 0.01, ns, not significant. Each experiment was independently performed two times, and the combined data shown.

Supplementary Figure-2

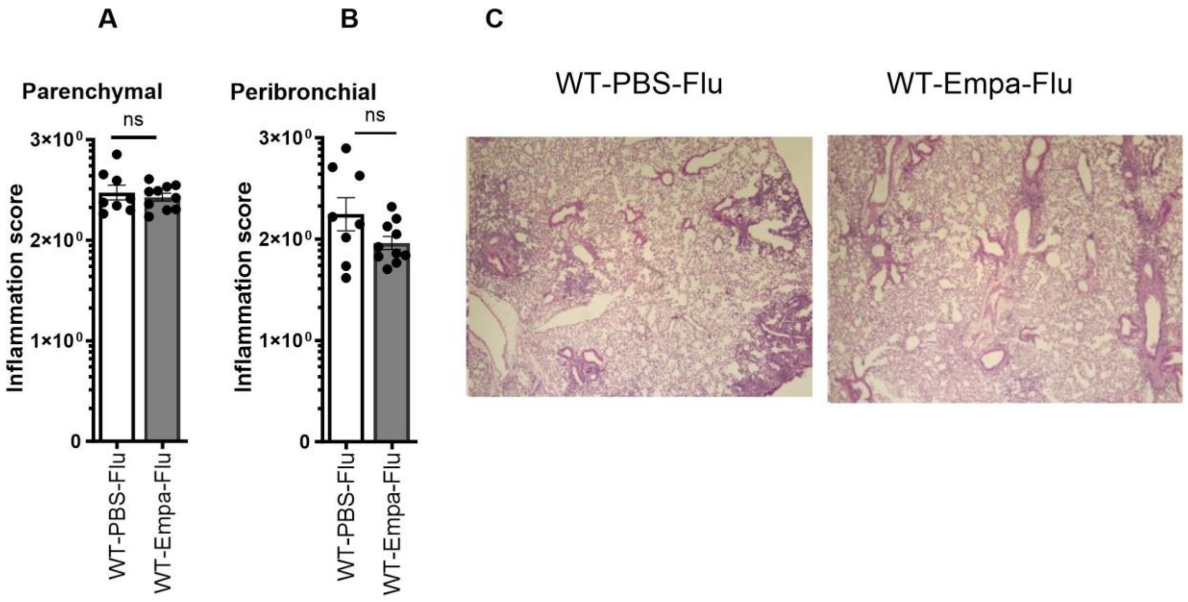

Supplementary Figure-2. Empagliflozin does not affect the inflammatory scores by histology.

C57BL/6 mice were treated with PBS or 10^3 PFU of influenza A PR/8/34 on day 0 and were treated with either DMSO vehicle or empagliflozin for 7 days. Parenchymal and Peri-bronchial inflammatory scores were analyzed by histology (A&B), and the representative pictures are shown (C). Data are represented as mean±SEM. Significance was tested by unpaired t-test. *p < 0.05, **p < 0.01, ns, not significant. Each experiment was independently performed two times, and the combined data shown.

Supplementary Figure-3

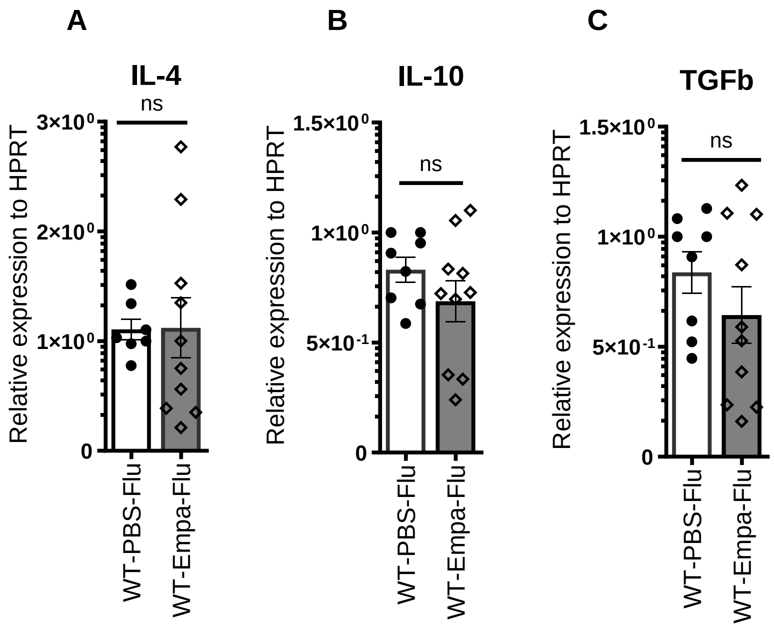

Supplementary Figure-3. Empagliflozin does not affect the expression of anti-inflammatory cytokines.

C57BL/6 mice were treated with PBS or 10^3 PFU of influenza A PR/8/34 on day 0 and were treated with either DMSO vehicle or empagliflozin for 7 days. Relative expressions of IL-4, IL-10, and TGFβ were measured by RT-PCR (A-C). Data are represented as mean±SEM. Significance was tested by unpaired t-test or one-way ANOVA. *p < 0.05, **p < 0.01, ns, not significant. Each experiment was independently performed two or more times, and the combined data shown.

Supplementary Figure-4

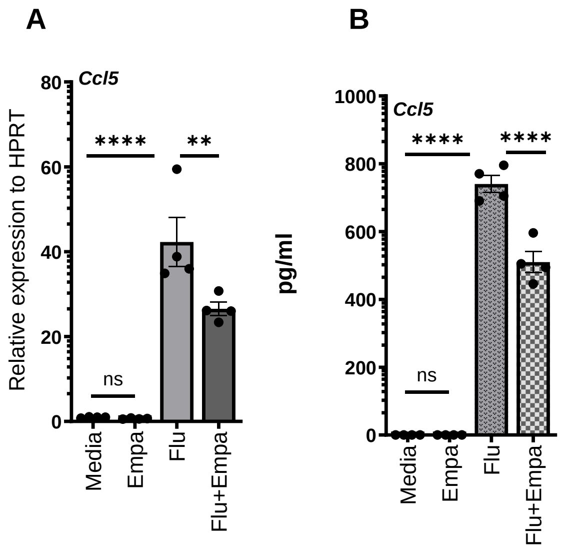

Supplementary Figure-4. CCL5 expression is decreased in lung epithelial C-10 cells treated with empagliflozin.

C10 cells were treated with PBS or 1 MOI of influenza A PR/8/34 and either DMSO vehicle or empagliflozin for 24 hours, the expression of CCL5 (A), and the levels of CCL5 were analyzed (B). Data are represented as mean±SEM. The cells were treated with triplicates or quadruplicates. Significance was tested by unpaired t-test or one-way ANOVA. $^{**}p < 0.01$, $^{***}p < 0.001$, $^{****}p < 0.0001$, ns-not significant. Each experiment was independently performed two or more times, and the representative data shown.
